# Heat Shock Proteins and Neurodegenerative Disorders

**DOI:** 10.1100/tsw.2008.48

**Published:** 2008-03-03

**Authors:** Eliza Ting-Li Soo, Yee-Kong Ng, Boon-Huat Bay, George Wai-Cheong Yip

**Affiliations:** Department of Anatomy, Yong Loo Lin School of Medicine, National University of Singapore, 4 Medical Drive, Block MD10, Singapore 117597, Singapore

**Keywords:** heat shock proteins, molecular chaperones, Alzheimer's disease, Parkinson's disease, Huntington's disease, prion disease

## Abstract

Heat shock proteins (HSPs) are evolutionarily conserved molecules and play important roles in fundamental cellular processes. They serve as molecular chaperones and hence provide a protective function in ensuring cell survival and repair of cellular damage after a stressful stimulus. This paper summarizes the current knowledge about the different roles of HSPs in aging and disease, focusing on the neurodegenerative disorders of Alzheimer's disease, Parkinson's disease, Huntington's disease, and prion disease.

## HEAT SHOCK PROTEINS

Heat shock proteins (HSPs), also known as stress proteins, were first discovered in 1962 by Ritossa[1], who observed that a transient increase in temperature induced puffing patterns in the chromosomes of salivary glands of *Drosophila melanogaster* larvae. Temperature increases were subsequently found to stimulate expression of proteins with molecular masses of 26 and 70 kDa[2]. These proteins were termed "heat shock proteins", as their expression was up-regulated in response to cellular insults, such as raised temperature, oxidative stress, chemicals, and irradiation[3]. Indeed, intracellular concentrations of HSPs are known to increase by approximately two- to threefold after cellular exposure to insults that induce protein misfolding or aggregation, or when there is a flux of newly synthesized non-native proteins[4].

HSPs are generally organized into different families according to their approximate molecular size or function – HSP100, HSP90, HSP70, HSP60, HSP40, and small HSPs (sHSPs) with molecular sizes ranging from 15 to 30 kDa[5]. They are present in the nucleus and the cytoplasm, as well as in cytoplasmic organelles, such as mitochondria and endoplasmic reticulum. Most HSPs function as molecular chaperones and help to maintain protein homeostasis within the cell. They provide protection against protein aggregation, facilitate the folding of newly synthesized polypeptides and refolding of proteins that have been damaged, and target and sequester proteins that have been severely damaged for degradation[4,6,7,8,9,10]. High molecular weight HSPs are known as ATP-dependent chaperones, assisting in the folding of newly synthesized or damaged proteins in an ATP-dependent active process, while sHSPs work in an ATP-independent manner[11]. A chaperone usually does not act alone, but is aided by other molecules, often other chaperones or several smaller cochaperones (such as HSP60 with HSP10, and HSP90 with HSP70)[12]. Although many proteins can fold to their native state spontaneously without the aid of any other molecular components *in vitro*, molecular chaperones are often required for protein folding to occur in a highly efficient manner *in vivo*[13].

## HSPs AND AGING

Various post-translational modifications occur within the lifespan of a stable protein[14]. These include oxidation, deamination, glycation, phosphorylation, acetylation, nitrosylation, and protein truncation from cleavage or deletion of N- or C-terminal residues[15,16]. Accumulation of misfolded proteins poses a danger to an aging cell. Since molecular chaperones such as HSPs are only capable of associating with misfolded proteins to prevent their aggregation, a more drastic mechanism, such as protein degradation, must be utilized in order to protect the cell from the potential toxicity of these proteins. Furthermore, induced expression of chaperones has been found to be impaired in the aged organism[17].

Protein degradation can occur by the action of proteasome, the key proteolytic apparatus, aided by various chaperones[8]. In the aged cell, decreased activity of the proteasome has been reported[18]. Moreover, glycated, oxidized, and aggregated proteins are often effective inhibitors of the proteasome[19,20]. Thus, accumulation of misfolded proteins coupled with a proteolytic apparatus with reduced effectiveness would result in a buildup of protein aggregates. These may then have harmful effects on cellular function and give rise to diseases associated with aging, particularly in neurons and other postmitotic cells[21].

## HSPs AND NEURODEGENERATIVE DISORDERS

An identifying characteristic of neurodegenerative disorders, such as Alzheimer’s disease (AD), Parkinson’s disease (PD), Huntington’s disease (HD), and Creutzfeldt-Jakob disease (CJD), is the formation of brain lesions possessing intracellular or extracellular deposits of protein aggregates[13,22]. These disorders are postulated to result from toxic effects of misfolded proteins[13] (Fig. 1).

### Alzheimer’s Disease

AD is late-onset dementia, presenting with progressive loss of memory, inability to recognize people and objects, and reduction in task performance. Neuronal degeneration is observed in the central nervous system, particularly in the basal forebrain and the hippocampus[23]. Neurofibrillary tangles (NFT) and β-amyloid (Aβ) plaques are defining features associated with AD. Microtubule-associated tau proteins are known to be hyperphosphorylated in AD, resulting in misfolding and formation of abnormal filaments, which eventually aggregate to produce NFTs[24]. Aβ is also involved in the pathogenesis of AD[25].

Elevated expression of the sHSPs αB-crystallin and HSP27/28, as well as of HSP70, has been reported in patients suffering from AD[26,27]. HSP27/28 and αB-crystallin may increase the neurotoxicity of Aβ 1-40 peptide by maintaining the peptide in a nonfibrillar, highly toxic form. On the other hand, sHSPs have also been reported to inhibit Aβ aggregation and effectively block the cerebrovascular toxicity of Aβ[28]. Both HSP70 and HSP90 facilitate solubilization of tau proteins and promote partitioning of tau into a productive folding pathway to form microtubules, thereby preventing aggregation of these proteins into NFTs. In addition, HSP70 and HSP90 are associated with accelerated tau degradation[24].

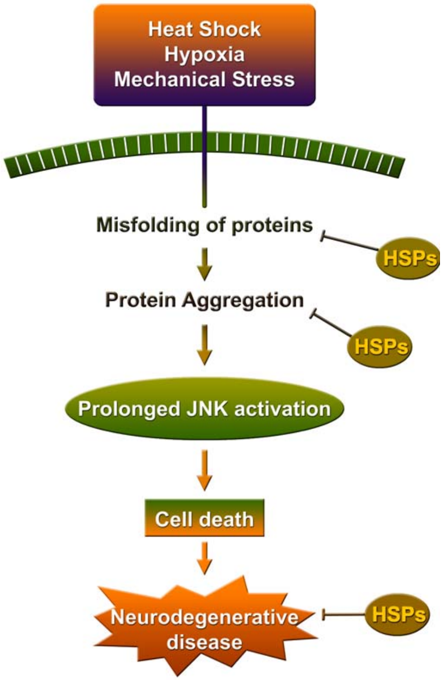

**FIGURE 1.** Misfolded proteins and neurodegenerative disorders. External stresses, such as heat shock, hypoxia, and mechanical stress, induce the production of reactive oxygen species and denaturation of cellular proteins. Moderated stress on the cell system induces HSP expression. These HSPs are capable of ensuring proper folding or treatment of misfolded proteins, as well as inhibiting protein aggregation, thus preventing neurodegenerative diseases that result from high stress–induced cell death[29].

## Parkinson's Disease

PD is an age-related, neurodegenerative movement disorder characterized by progressive degeneration of dopaminergic neurons in the substantia nigra. The hallmark of PD is accumulation of α-synuclein (AS), the main component of Lewy bodies in midbrain dopaminergic neurons[29]. Examination of the aggregated proteins in Lewy bodies of PD patients showed the presence of several HSPs[30]. In addition, patient glial cells have been reported to express αB-crystallin. Coexpression of HSP70 with AS has been shown to inhibit the formation of AS fibrils and reduce their toxicity, both *in vitro* as well as *in vivo* in *Drosophila* and mouse, possibly by binding of HSP70 to prefibrillar AS[31,32,33]. Indeed, Huang et al. have demonstrated that this inhibition occurs through the binding of HSP70 with various intermediate structures[34]. Dietary restriction has been shown to induce HSP70 and Grp78 expression, resulting in protection against PD[35].

## Huntington's Disease

HD is a progressive, neurodegenerative disorder resulting from expansion of a CAG repeat that codes for polyglutamine in the N-terminus of the huntington protein. It is characterized by selective neuronal loss, primarily in the cortex and striatum. This results in alterations in movement, memory, and mood[36]. Polyglutamine repeats make proteins more vulnerable to aggregation. A significant feature of polyglutamine diseases is the formation of neuronal inclusions that consist of insoluble, granular, and fibrous deposits[36]. Increased levels of HSP40, HSP60, HSP70, and HSP100 have been shown to inhibit polyglutamine-induced protein aggregation and thus impede disease progression[8,37,38,39,40].

## Prion Diseases

The central feature of prion diseases is the aggregation of pathologic prion proteins, such as $PrP^{Sc}$, an abnormal isoform of the cellular prion protein[41]. Prion has been held responsible for a number of degenerative brain diseases, including CJD in man, bovine spongiform encephalopathy in cows, and scrapie in sheep. The key event in CJD is the extracellular deposition of aggregates of $PrP^{Sc}$ in the brain of affected individuals. It is thought that cellular toxicity is conferred either by $PrP^{Sc}$ aggregates or, more likely, by the process of their formation[13]. In scrapie, amyloidogenic proteins such as $PrP^{Sc}$ have been postulated to cause neuronal injury and, subsequently, death. Elevated HSP72 levels have been reported in astrocytes, which may have been produced to act as a molecular chaperone to prevent the formation or deposition of neurotoxic proteins, and also to target those proteins for degradation[42]. Interestingly, studies have shown that bovine cellular prion protein $PrP^{c}$ is able to bind to αB-crystallin. However, it is not known if this association can prevent the formation of PrP fibrils and neurotoxicity[43].

## CONCLUSION

HSPs are essential molecular chaperones in the cell, functioning to preserve the correct conformation of proteins and also to repair misfolded proteins. As discussed above, a large body of evidence supports the involvement of HSPs in neurodegenerative disorders. Understanding the various mechanisms involved in the formation of inclusion bodies in the pathophysiological development of neurodegenerative disorders would aid in the quest for therapeutic alternatives. Knowledge of the roles played by HSPs in protein aggregation and subsequent toxicity may lead to novel treatment approaches to neurodegenerative disorders by targeting the fate of misfolded proteins.
